# Perceived Readiness and Ability to Socially Distance During the Early COVID-19 Epidemic in a U.S. Metropolitan Area: Implications for Local Public Health Preparedness

**DOI:** 10.3390/epidemiologia7020048

**Published:** 2026-04-02

**Authors:** Emmanuel K. Tetteh, Julia D. López, Collin McGovern, Gifty Aboagye-Mensah, Elvin H. Geng, Virginia R. McKay

**Affiliations:** 1 School of Public Health, Washington University, St. Louis, MO 63130, USA; 2 Division of Infectious Diseases, Department of Medicine, Washington University School of Medicine, St. Louis, MO 63110, USA

**Keywords:** COVID-19, social distancing, mixed methods, socio-ecological model, risk perception, public health policy

## Abstract

Background/Objectives: Nonpharmaceutical interventions such as social distancing and face mask use were central to controlling infectious disease transmission during the early phases of the COVID-19 pandemic, particularly when vaccines and treatments were limited or unevenly available. Although public health strategies emphasized individual compliance, adherence varied widely. Empirical evidence remains limited regarding how individuals integrate influences across individual, interpersonal, and community levels when assessing their ability and readiness to socially distance. This study examined how residents evaluated, prioritized, and experienced multi-level factors shaping perceived ability and readiness to practice social distancing during the early phase of the COVID-19 epidemic. Methods: We conducted a cross-sectional online survey of adults (≥18 years) residing in St. Louis City and St. Louis County, Missouri, between April and July 2020. Participants selected and ranked individual/interpersonal and community-level factors influencing social distancing and provided open-ended explanations of their choices. Quantitative data were analyzed descriptively to assess selection frequency and ranking priority. Qualitative responses were analyzed using iterative thematic coding to examine how participants interpreted and combined these factors. Results: The analytic sample included 1692 respondents. At the individual/interpersonal level, family and friends’ distancing behavior (58.9%), desire for in-person interaction (52.4%), and personal risk of COVID-19 (48.9%) were frequently selected, while personal risk, caring for others, and ability to work from home were most often ranked as the highest priority. At the community level, others’ distancing in public spaces (66.2%), availability of COVID-19 testing (58.9%), and businesses’ ability to ensure distancing and sanitation (57.2%) were most frequently selected, with epidemic severity, testing availability, and treatment availability ranked as most influential. Qualitative findings indicated that respondents experienced these influences as interconnected, integrating personal and relational risk, local epidemic conditions, healthcare access, visible community norms, and employer policies. Conclusions: Perceived ability and readiness to practice social distancing emerge from interdependent social and structural conditions rather than isolated individual motivations. Public health responses to emerging infectious diseases may be more effective when individual-level guidance is complemented by accessible testing and treatment, supportive workplace policies, and community environments that visibly reinforce protective behaviors.

## 1. Introduction

The COVID-19 pandemic highlighted the critical role of nonpharmaceutical interventions (NPIs) such as social distancing, face mask use, and hand hygiene in infectious disease prevention [1]. While vaccines demonstrated high effectiveness in reducing hospitalization and death [2], their effectiveness against infection and onward transmission was more limited, particularly with the emergence of highly transmissible variants [3]. In this context, NPIs remained essential components of transmission control, especially when community mitigation measures were relaxed [3]. Vaccines and NPIs therefore serve complementary roles in epidemic management. In retrospect, adherence varied widely over time and across populations [4,5], emphasizing the need to understand the social, interpersonal, and structural conditions that shape individuals’ perceived ability and readiness to adopt protective behaviors.

A substantial body of research has examined barriers and facilitators to social distancing during COVID-19, identifying factors such as perceived personal risk [6], trust in public health guidance [5], ability to work remotely [7], and social norms within families and peer groups [4]. Studies have also highlighted the influence of broader community and system-level conditions [8,9,10], including policy mandates, testing availability, healthcare capacity, and workplace practices. Importantly, the ability to adhere to social distancing was not evenly distributed across populations [4,5,6,7,8,9]; structural determinants such as income, occupation, housing conditions, and access to healthcare shaped individuals’ capacity to comply, contributing to inequities in exposure risk and health outcomes. Collectively, this literature demonstrates that social distancing is shaped by influences operating at multiple levels of the social ecology.

However, two important gaps remain. First, while existing research has identified a range of individual, interpersonal, and structural influences on social distancing, much of this evidence treats these influences as discrete or additive, emphasizing whether particular factors are present rather than how individuals prioritize them when making real-world decisions. Second, relatively few studies integrate quantitative assessments of factor importance with qualitative evidence capturing how people interpret and combine these influences in practice. As a result, it remains unclear how individuals weigh personal risk, protection of close others, community conditions, and institutional support simultaneously when assessing their readiness and ability to socially distance [4].

The socio-ecological model provides a useful framework for examining multi-level influences on health behavior, positing that individual actions are shaped by interpersonal relationships, community contexts, and broader structural conditions [11]. While widely applied in public health research, empirical studies during COVID-19 often operationalized these levels separately [12,13], offering limited insight into how individuals experienced the interaction of these influences in everyday decision-making. Understanding how people navigated these overlapping considerations during the pandemic is particularly relevant for informing future epidemic preparedness, including the design of local response strategies, healthcare system readiness, and public communication efforts [14].

To address these gaps, we conducted a mixed-methods study of adults residing in St. Louis City and County, Missouri, USA, during the early phase of the regional COVID-19 epidemic. The St. Louis metropolitan area is characterized by substantial socioeconomic, racial, and jurisdictional diversity, providing a valuable context for examining how multi-level influences shape social distancing behaviors. This study aimed to examine how individuals assessed and prioritized multi-level influences on perceived readiness and ability to socially distance during an emerging epidemic. This study contributes empirical evidence to inform understanding of behavioral responses during public health emergencies and to guide local preparedness and response strategies for future infectious disease threats.

## 2. Materials and Methods

### 2.1. Study Design and Setting

We conducted a cross-sectional mixed-methods study to examine factors influencing perceived readiness and ability to practice social distancing during the COVID-19 epidemic. Data were collected via an online survey administered to adult residents of St. Louis City and St. Louis County, Missouri, between 23 April and 2 July 2020. The study employed a convergent mixed-methods design in which quantitative ranking data and qualitative open-ended responses were collected concurrently and integrated at the interpretation stage. The Institutional Review Board at Washington University in St. Louis reviewed and approved the study procedures and determined the study to be exempt. Accordingly, signed informed consent was not required; participants were provided with an exempt information sheet to ensure their informed participation prior to the study.

### 2.2. Study Population and Recruitment

Individuals aged 18 years or older who resided in St. Louis City or St. Louis County were eligible to participate. Participants were recruited through targeted social media advertising and distribution via local organizational email listservs. Participants were not given an incentive for participation; however, for every individual who participated in the survey, a $1 donation was made to a local non-profit working to counter the economic impacts of the epidemic in the St. Louis Region, receiving up to $2000. Recruitment occurred continuously throughout the data collection period. While the sampling strategy was non-probabilistic, recruitment efforts aimed to achieve diversity across age, gender, socioeconomic status, and race and ethnicity.

### 2.3. Survey Instrument and Measures

The survey was administered anonymously using Qualtrics survey software (Qualtrics, Provo, UT, USA) and included questions across four domains: (1) participant characteristics; (2) social distancing behaviors; (3) factors influencing social distancing; and (4) information sources used to guide distancing decisions, which are published elsewhere [5]. The survey instrument is included as Supplementary File S1. The current analyses focused on factors influencing social distancing, which were operationalized using constructs derived from the socio-ecological model, encompassing individual, interpersonal, and community-level influences on health behavior [11].

Given the limited COVID-19-specific evidence available in early 2020, factor selection was guided by theory and by evidence from analogous epidemic contexts, with attention to relevance for social distancing behavior. Candidate factors were identified through a review of the published literature available at the time of survey development, including established research on nonpharmaceutical interventions [15], risk perception [16], and protective behaviors during prior infectious disease outbreaks (e.g., influenza, SARS, and Ebola) [17,18,19]. The survey instrument was reviewed by members of the study team with expertise in public health, implementation science, and infectious diseases for clarity and face validity prior to fielding; given the urgent timeline of early pandemic data collection, formal pilot testing was not conducted.

To reduce respondent burden, factors were grouped into two domains: (1) individual- and interpersonal-level factors and (2) community-level factors. Participants were asked to select up to 10 factors within each domain that they perceived as influencing their ability and readiness to practice social distancing. Participants then ranked their selected factors in order of perceived importance. This rank-order approach was used to capture not only whether a factor was influential, but also its relative priority in decision-making, recognizing that individuals often navigate multiple, competing influences simultaneously. Participants were additionally provided with the option to submit open-ended responses describing how and why the selected factors influenced their social distancing behavior.

### 2.4. Data Analyses

#### 2.4.1. Quantitative Data

Quantitative analyses were conducted using the R statistical environment [20]. Participant characteristics and factor selection frequencies were summarized using descriptive statistics. Missing data were minimal (<5% across variables) and were handled using complete case analysis for descriptive summaries and ranking analyses. To assess the relative priority of influencing factors, ranking data were analyzed using a priority-weighted approach [21]. For each factor, rankings were summarized by frequency of selection and by distribution of rank positions, allowing for identification of factors most frequently prioritized as highly influential. This approach emphasizes relative importance rather than assuming equal influence across selected factors.

To examine subgroup variation in prioritization patterns, we conducted additional multivariable logistic regression analyses. For selected factors most frequently ranked as highest priority within the individual/interpersonal and community domains, we created binary outcomes indicating whether a respondent ranked a given factor as their top priority. Separate logistic regression models were estimated for each selected factor to assess associations between respondent characteristics (age, gender, race/ethnicity, income, comorbidity status, and employment status) and the likelihood of ranking that factor as most influential. Adjusted odds ratios (aORs) and 95% confidence intervals (CIs) were calculated. Due to sparse data in some response categories, select demographic variables were collapsed to ensure model stability. Analyses were conducted using complete case data.

#### 2.4.2. Qualitative Data

Open-ended responses from the survey were analyzed using an inductive thematic analysis approach [22]. Initial coding categories were informed by the most frequently selected and highly ranked factors in the quantitative analysis, allowing qualitative findings to elaborate and contextualize quantitative priorities. An iterative codebook was developed through multiple rounds of coding and refinement. The codebook and code summaries are included as Supplementary File S2. Two authors independently coded a subset of responses (~15%) to assess consistency in interpretation, with discrepancies resolved through discussion. Coding then proceeded across the full dataset. Rather than treating themes as discrete or mutually exclusive, the analysis focused on identifying patterns in how participants described the interaction and co-occurrence of influences across individual, interpersonal, and community levels. Conceptual saturation was assessed based on the recurrence and coherence of themes rather than the volume of responses.

## 3. Results

A total of 3180 individuals initiated the survey, of whom 1692 met eligibility criteria (aged ≥ 18 years and residing in St. Louis City or County) and were included in the analytic sample. Most respondents identified as women (75.8%), and 63.0% were between 18 and 45 years of age. Over half of participants (58.0%) reported annual household incomes of at least $70,000, and 70.3% reported no COVID-19–related comorbid conditions. Nearly all respondents (97.0%) reported residing in an area with a social distancing mandate at the time of data collection. Additional demographic and socioeconomic characteristics are summarized in Table 1.

### 3.1. Prioritization of Individual and Interpersonal Level Factors

**Figure A1:**
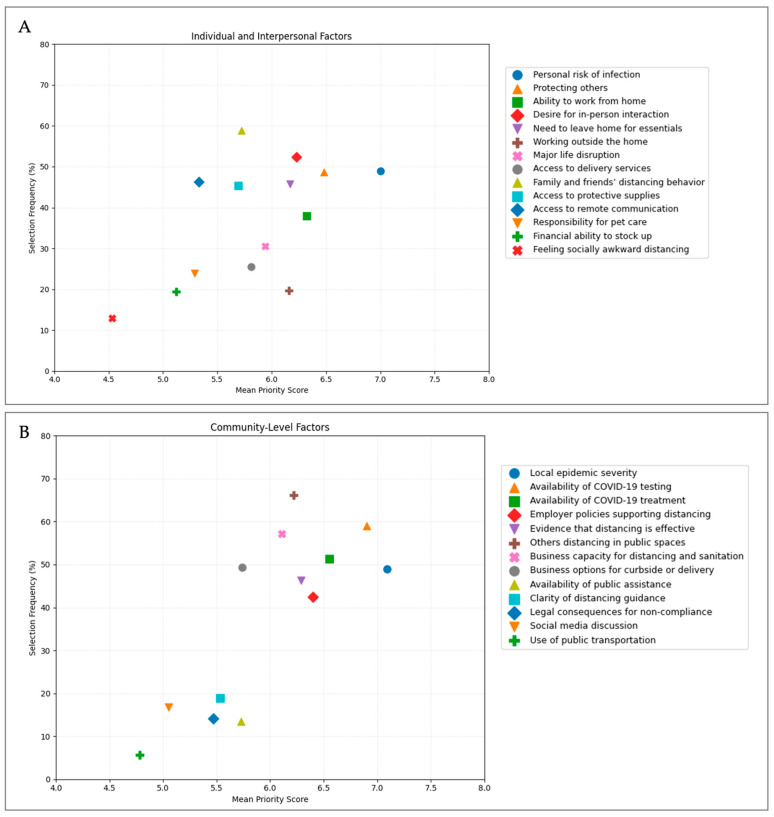
Selection frequency and mean weighted priority score for factors influencing social distancing. (Panel (**A**) displays individual and interpersonal factors, and Panel (**B**) displays community-level factors).

Participants selected and ranked factors they perceived as influencing their ability and readiness to practice social distancing (Appendix A Figure A1). On average, respondents selected six individual/interpersonal factors (range: 1–14), and nearly all ranked each selected factor, indicating engagement with the prioritization task. The most frequently selected individual/interpersonal factors were family and friends practicing social distancing (58.9%), desire for in-person interaction (52.4%), personal risk of COVID-19 infection (48.9%), and caring for others important to them (48.7%).

However, selection frequency alone did not fully reflect perceived importance. When rankings were incorporated using a weighted priority approach, a different pattern emerged. Personal risk of COVID-19 infection received the highest mean weighted priority score (7.00) and was most frequently ranked as the single most influential factor. Caring for others was important to the respondent (mean score: 6.48), and the ability to work from home (6.32) was also consistently prioritized highly. In contrast, some factors that were commonly selected, such as family and friends practicing social distancing, and availability of video chat and internet, received lower weighted priority scores, indicating that while these factors were relevant to many respondents, they were less often viewed as decisive influences on behavior. Priority distributions for individual and interpersonal factors are presented in Table 2.

### 3.2. Prioritization of Community-Level Factors

At the community level, respondents selected an average of six factors (range: 1–13). The most frequently selected factors were others practicing social distancing in public spaces (66.2%), availability of COVID-19 testing (58.9%), businesses’ ability to ensure distancing and sanitation (57.2%), and availability of COVID-19 treatment (51.3%).

When rankings were incorporated using a weighted priority approach, the severity of the COVID-19 epidemic where respondents lived or worked emerged as the most influential community-level factor, receiving the highest mean weighted priority score (7.09) and most frequently being ranked first. Availability of COVID-19 testing (6.90) and availability of treatment (6.55) were also consistently prioritized highly. Factors reflecting institutional and healthcare system capacity, such as employer policies supporting social distancing and evidence that social distancing was working, received moderate to high priority scores. Detailed selection frequencies and weighted priority scores for community-level factors are presented in Table 3.

### 3.3. Subgroup Variation in Prioritization of Highest-Ranked Factors

Multivariable logistic regression analyses (Supplementary File S3, Tables S1–S6) indicated that prioritization patterns varied by respondent characteristics. Adults aged 46 years and older had higher odds of ranking caring for others (aOR 1.59, 95% CI 1.12–2.27) and personal risk (aOR 1.62, 95% CI 1.18–2.22) as their top priority, but lower odds of prioritizing working from home (aOR 0.56, 95% CI 0.35–0.89). Respondents without comorbid conditions were less likely to rank personal risk as most influential (aOR 0.48, 95% CI 0.30–0.77). White respondents had lower odds of prioritizing personal risk (aOR 0.61, 95% CI 0.41–0.92) and epidemic severity (aOR 0.53, 95% CI 0.32–0.87) compared to BIPOC respondents. Men were more likely to rank epidemic severity as the highest priority (aOR 1.66, 95% CI 1.06–2.62), and unemployed respondents were more likely to prioritize treatment availability (aOR 1.75, 95% CI 1.02–2.99). No consistent subgroup differences were observed for testing availability.

### 3.4. Qualitative Themes Explaining Prioritization Patterns

Of the analytic sample, 156 respondents (9.2%) provided open-ended explanations describing how selected factors influenced their social distancing behavior. Thematic analysis identified four interrelated themes that explained how respondents integrated multiple influences when assessing readiness and ability to socially distance.

#### 3.4.1. Theme 1: Weighing Personal Risk in the Context of Testing, Treatment, and Epidemic Severity

Respondents frequently framed personal risk not as an isolated characteristic, but as contingent on local epidemic severity and the perceived availability of testing and treatment. Many described their willingness to maintain strict social distancing as heightened in contexts where testing was limited, treatment options were uncertain, or healthcare systems appeared strained. In these accounts, distancing was described as a protective strategy to avoid both personal infection and additional burden on healthcare workers (Table 4, quotes 1–6). Several respondents also explicitly linked future willingness to relax distancing behaviors to improvements in testing access or treatment availability, regardless of changes in formal policy. Even in the presence of stay-at-home order rollbacks, respondents described continuing to distance in the absence of confidence in the healthcare system’s capacity to detect and manage cases (Table 4, quotes 3–5). Others distinguished between factors they viewed as essential for safety, such as epidemic severity and treatment availability (Table 4, quote 8), and factors that made distancing more tolerable but did not fundamentally alter risk, such as curbside pickup or delivery options (Table 4, quote 7).

#### 3.4.2. Theme 2: Heightened Responsibility to Protect High-Risk Household Members and Close Contacts

Respondents commonly described social distancing as a moral and relational obligation tied to protecting vulnerable household members and close contacts. Even when individuals perceived their own risk of severe illness as low, the presence of high-risk family members, such as older adults, individuals undergoing cancer treatment, or those with chronic conditions, substantially elevated the perceived necessity of distancing (Table 4, quotes 9–12). In these narratives, distancing decisions were framed less as personal choices and more as responsibilities embedded in caregiving roles and family relationships. Respondents expressed fear of becoming a vector of transmission and emphasized the emotional consequences of potentially causing harm to loved ones. This sense of responsibility often intensified adherence and reduced tolerance for risk-taking, regardless of broader community behavior or policy changes.

#### 3.4.3. Theme 3: Dependence on Community Norms and Public Behavior

Many respondents described their ability to socially distance as shaped by the behavior of others in public spaces and the extent to which community norms supported protective practices. Observations of crowding, improper mask use, or lack of enforcement by businesses undermined respondents’ sense of safety and prompted avoidance of public settings altogether (Table 4, quotes 13–16). Respondents emphasized that distancing was more difficult, and sometimes emotionally taxing, when others failed to comply, particularly in essential settings such as grocery stores. At the same time, visible adherence by others was described as reassuring, normalizing, and reinforcing distancing behavior, reducing feelings of awkwardness or social isolation (Table 4, quotes 17–18). These accounts highlight how community behavior functioned both as a practical constraint on distancing and as a social signal shaping comfort and confidence.

#### 3.4.4. Theme 4: Structural Dependence on Employer Policies and Institutional Support

Employment conditions emerged as a relevant structural determinant of respondents’ ability to practice social distancing. Participants repeatedly described their willingness to distance as constrained or enabled by employer policies, particularly the availability of remote work (Table 4, quotes 19–23). For some, the ability to work from home was described as their determining factor supporting sustained distancing. Respondents also expressed frustration and anxiety about the lack of control they had over workplace decisions, especially when employers prioritized a return to in-person work despite perceived ongoing risks and limited testing capacity (Table 4, quotes 21, 24–26). For individuals in jobs that required physical presence, distancing was described as fundamentally incompatible with employment demands, reinforcing a sense of vulnerability and lack of choice (Table 4, quote 27).

## 4. Discussion

The COVID-19 epidemic provides an opportunity to understand factors that influence a person’s readiness and ability to engage in social distancing behaviors to help prevent disease spread. Using a mixed-methods approach that combined ranked quantitative data with qualitative explanations, we found that social distancing decisions were shaped by integrated judgments across individual, interpersonal, and community levels rather than by isolated influences. Taken together, these findings suggest that social distancing operates as a collective, system-dependent behavior rather than a purely individual choice. Importantly, perceived readiness does not necessarily equate to actual ability. Several respondents expressed strong motivation to socially distance but described structural constraints, such as employment requirements, that limited their capacity to do so. This distinction may be especially salient among socioeconomically vulnerable populations, for whom structural barriers constrain behavioral choice despite high perceived risk.

Our findings are broadly consistent with prior studies showing that perceived risk, concern for others, and capacity to work remotely are central to social distancing behavior during COVID-19 [6,7,23]. However, the use of ranked prioritization data extends this literature by demonstrating that not all commonly cited factors are equally influential in decision-making. While many participants identified a wide range of factors as relevant to their circumstances, only a subset, particularly personal and relational risk and structural capacity to distance, consistently emerged as decisive. This distinction between contextual relevance and behavioral priority has been largely absent from prior survey-based research and may help explain why interventions that address known “barriers” do not always translate into sustained behavior change.

In supplementary analyses, prioritization patterns varied across demographic and structural characteristics. Older adults more strongly emphasized personal and relational risk, whereas younger respondents were more likely to prioritize structural work-related constraints. Racial differences in prioritization of personal risk and epidemic severity suggest that perceived vulnerability and community context may shape how individuals weigh protective considerations. Employment status also influenced prioritization, particularly with respect to remote work and treatment availability. Although these subgroup differences were secondary to our primary analyses, they highlighted that readiness and ability to socially distance are embedded within demographic position and structural circumstance rather than reflecting uniform individual preferences.

Qualitative findings further situate these priorities within lived experience and clarify how individuals interpreted and combined influences across levels of the socio-ecological model. In contrast to models that frame social distancing primarily as an individual risk-management strategy [13,23,24], participants frequently described distancing as a relational and moral practice oriented toward protecting vulnerable household members and avoiding strain on healthcare systems. These accounts align with prior work emphasizing altruism and collective responsibility during infectious disease outbreaks [17,18,19], while also emphasizing the importance of perceived system capacity, particularly access to testing and treatment, in shaping willingness to maintain restrictive behaviors.

These findings have important implications for public health preparedness and response. Much of the early public health messaging during COVID-19 emphasized individual responsibility and personal risk reduction [25]. While such messaging is necessary, the results of this study suggest it may be insufficient when not accompanied by supportive community and institutional conditions. For example, local preparedness plans could include pre-established employer guidance frameworks that incentivize remote work where feasible, and publicly accessible dashboards communicating testing capacity and healthcare system strain. Such structural support may enhance both willingness and practical capacity to adhere to nonpharmaceutical interventions. Accessible testing and treatment, employer policies that enable remote work or ensure safer in-person environments, and consistent enforcement of distancing practices in public spaces appear to be critical in enabling individuals to act on their motivation to distance.

The study also offers insight into how local healthcare preparedness and alert systems shape behavioral readiness. Participants’ emphasis on testing availability, treatment access, and local epidemic severity indicates that perceptions of system capacity and responsiveness function as key signals guiding protective behavior. Transparent communication about healthcare resources, timely dissemination of local epidemic indicators, and visible reinforcement of protective norms may help translate public health guidance into sustained action [8,26]. Integrating behavioral guidance with demonstrable system readiness may therefore be essential for maintaining nonpharmaceutical interventions over time.

Our findings also align with broader themes emphasized by other scholars in healthcare preparedness and alert systems, particularly the importance of health systems’ resilience and local preparedness in epidemic response. Consistent with recent work highlighting the role of institutional capacity, healthcare system response, and surveillance in managing infectious disease threats [27,28], our results suggest that individuals calibrate protective behaviors in relation to perceived healthcare access, institutional support, and visible community adherence. These findings reinforce the importance of aligning behavioral guidance with demonstrable system preparedness in future epidemic planning.

Several limitations should be considered when interpreting these findings. The study relied on a non-probabilistic sample drawn from a single metropolitan area, which may limit generalizability to other settings. Measures relied on self-reported perceptions of readiness and ability, which may be influenced by recall bias or social desirability bias and may not fully reflect actual behavior. Data were collected during the early phase of the COVID-19 epidemic, and perceptions of social distancing may have shifted as vaccines, treatments, and policies evolved. In addition, qualitative responses were provided by a subset of participants and were intended to offer explanatory depth rather than population-level inference. Despite these limitations, the convergence between quantitative prioritization patterns and qualitative themes strengthens confidence in the study’s central insights.

## 5. Conclusions

This study demonstrates that perceived readiness and ability to practice social distancing are shaped by interdependent individual, interpersonal, and community-level conditions that operate simultaneously. Public health responses to emerging infectious diseases may benefit from moving beyond approaches that focus solely on individual behavior and instead investing in social and structural support that enables and normalizes protective actions at the population level. Such an approach is likely to be critical for strengthening local preparedness and improving the effectiveness of nonpharmaceutical interventions in future epidemics.

## Figures and Tables

**Table 1:** Demographic Characteristics of Study Population (N = 1692).

Category	Characteristic	N	%
County of Residence	St. Louis City	789	46.6
	St. Louis County	903	53.4
Age	18–25	168	9.9
	26–35	430	25.4
	36–45	463	27.4
	46–55	300	17.7
	55–65	330	19.5
Gender	Women	1279	75.8
	Men	355	21.0
	Non-binary	40	2.4
	No response	13	0.8
Race/ethnicity	BIPOC ^+^	263	16.0
	White alone	1425	86.9
	No response	4	0.2
Annual Household income	Less than $40,000	236	13.9
	$40,000 to less than $70,000	325	19.2
	$70,000 to less than $100,000	292	17.3
	$100,000 or more	687	40.6
	No response	152	9.0
Work status	Working from home	933	55.1
	Working outside the home	261	15.4
	Furloughed	139	8.2
	Unemployed	333	19.7
	No response	26	1.5
Health Insurance Status	Medicare, Medicaid or National Health Insurance	146	8.6
	Employer-sponsored, Private or Group	1222	72.2
	Veteran Affairs or Military	72	4.3
	No Insurance	60	3.5
	No response	26	1.5
Comorbid Conditions	Asthma and Chronic respiratory conditions	162	9.6
	Cancer and Immunosuppressive conditions	52	3.1
	Cardiovascular conditions	88	5.2
	Other conditions *	165	9.8
	None of the above	1190	70.3
Social Distancing Mandate	Present	1641	97.0
	Absent	28	1.7
	Not sure	23	1.3

^+^ BIPOC: American Indian or Alaskan Natives, Asian, Black, Hispanic, or LatinX. * Other conditions include chronic kidney disease, diabetes, and multiple comorbidities.

**Table 2:** Individual and Interpersonal Level Factors Influencing Social Distancing: Selection Frequency and Priority.

Factor	Selected, n (%)	Mean Weighted Priority Score †
My own personal risk of getting COVID-19	828 (48.9)	7
Caring for others who are important to me	824 (48.7)	6.48
Working from home	641 (37.9)	6.32
Wanting to interact with others in person	886 (52.4)	6.23
Needing to leave for healthcare or to buy things I need	775 (45.8)	6.17
Continuing to work outside my home	333 (19.7)	6.16
A major life event (e.g., moving, family death)	517 (30.6)	5.94
Having groceries and goods delivered	431 (25.5)	5.81
My family and friends practicing social distancing	997 (58.9)	5.72
Having supplies like disinfecting wipes or hand sanitizer	767 (45.3)	5.69
Having video chat and internet to interact remotely	784 (46.3)	5.33
Caring for pets	404 (23.9)	5.29
Having enough money to make bulk purchases	328 (19.4)	5.12
It feels socially awkward	218 (12.9)	4.53

† Mean priority score calculated using weighted rankings (rank 1 = 10 through rank 10 = 1) among respondents who selected each factor; higher scores indicate higher perceived priority.

**Table 3:** Community Level Factors Influencing Social Distancing: Selection Frequency and Priority.

Factor	Selected, n (%)	Mean Weighted Priority Score †
The severity of the COVID-19 epidemic where I live or work	827 (48.9)	7.09
The availability of testing for COVID-19 where I live or work	998 (59.0)	6.9
The availability of treatment for COVID-19	868 (51.3)	6.55
My employer has policies to support social distancing	718 (42.4)	6.4
Evidence showing that social distancing is working	783 (46.3)	6.29
Others practicing social distancing in public spaces	1120 (66.2)	6.22
Businesses’ ability to ensure distancing and sanitation	968 (57.2)	6.11
Businesses offering curbside pick-up or delivery	834 (49.3)	5.74
Public assistance to help cover my needs	228 (13.5)	5.73
Clarity about how to practice social distancing	319 (18.9)	5.53
Legal consequences of not practicing social distancing	239 (14.1)	5.47
What others are saying on social media	284 (16.8)	5.05
Using public transportation	94 (5.6)	4.78

† Mean priority score calculated using weighted rankings (rank 1 = 10 through rank 10 = 1) among respondents who selected each factor; higher scores indicate higher perceived priority.

**Table 4:** Qualitative themes and representative quotes.

*Weighing Personal Risk in the Context of Testing, Treatment, and Epidemic Severity*
(1) “Until there is sufficient, accurate testing & treatment for COVID, I am willing to stay home & help flatten the curve. There is no need to add to the overwhelming task that our frontline workers are currently dealing with.” (2) “No one has notified of any testing available, and we don’t know who to call. I am over 63 years and my husband is 78. Hearing about the severity of symptoms I am concerned about getting the virus. I believe in the “right to try”. I have heard a person call in to the local radio station, of being sent home and not treated for the illness, only to have to return to the emergency room because they could not get his breath. Ultimately, he was treated with hydroxychloroquine and recovered. He is younger than I am. That vaccine can’t come fast enough. We have children and grandchildren we would like to visit (on each coast) but are afraid to travel.” (3) “Testing is the single most important factor for our family to begin to drop our social distancing protocol. Next would be treatment. Even if stay at home orders are not extended, we do not intend to modify our behavior.” (4) “I don’t trust easing of social distancing measures if we don’t have adequate testing and treatment in place.” (5) “We need better testing and treatment. The stay-at-home orders need to end soon. People will stop following if some restrictions aren’t loosened.” (6) “My willingness and ability to practice social distancing is increased by the aforementioned factors. COVID-19 is spreading quickly in the St. Louis area, and I don’t want to get or be a carrier so I stay home other than going to work.” (7) “There seems to be a difference between the things that make social distancing absolutely imperative vs the things that make social distancing more comfortable. Like, there severity of the outbreak and the availability of treatment are critical factors, whereas the availability of curbside just makes it emotionally easier.” (8) “I think the severity of the outbreak, and how easy it is to get tested and treated would make me more cautious, but even a small outbreak could spread quickly without controls--mainly distancing. If there is a strong, clear, consistent message that everyone understands and I see people following it when I am out--basically top-down orders--I can see myself doing what I am told to do much better, sad to say. Guess I am a follower.”
*Heightened Responsibility to Protect High-Risk Household Members and Close Contacts*
(9) “I know what do, and I’m willing to keep social distance to protect others, especially my wife who is at a high risk for complications. But I worry about the lack of concern of people who protest that these measures impose on their rights. They fail to realize their responsibility to maintain health in the community, and they scare me because they don’t care for anyone other than themselves.” (10) “My daughter is a type one diabetic. We are very motivated to keep her away from the virus until a better treatment is available” (11) “I just don’t want to go back and work in a clinic where staff and patients are not wearing masks and we work in a small room. This terrifies me. My mom is going through chemo and I need to visit her/bring groceries—I’m staying far away but this is priority and I’d never forgive myself if I brought her something.” (12) “I am very concerned about other people getting my family sick, and us spreading it to my family member with stage 4 cancer. He lives alone so my husband is one of his main caregivers. We have no choice other than to take the most precautions necessary.”
*Dependence on Community Norms and Public Behavior*
(13) “What impacts my ability to perform social distancing is when I go to stores and employs are not wearing masks. Or customers (also not wearing masks) violate the 6ft distance rule. For instance, yesterday I went to [a local store] for paint. It was packed. I won’t go back. I’ll order online instead. Even after some of the rules are lifted in the area, we will continue to frequent businesses that offer curb-side pick-up/delivery until treatments, or a vaccine is available.” (14) “I don’t really trust other’s dedication; I see incorrectly wearing of masks (nose exposed) so I would rather stay home than swim with idiots.” (15) “I have not been in a grocery store for 7 weeks now because the last time I did it was terrifying. Blatant disregard for social distancing. I was running away from the old ladies FOR THEIR SAKE. And my dad works in a supermarket, so I’m scared to death for him. The fewer people coming & going in those doors, the better. We’ve been ordering carryout from restaurants about twice a week (probably a bit more than normal for us) because we want them to survive with us.” (16) “I feel fairly comfortable with my ability to reduce my risk and that of my family; however, between working outside the home and leaving the house for essential errands, I worry about the increased risk due to *other* people not following social distancing and good hygiene guidelines.” (17) “I don’t like being seen as hysterical so seeing no one else in CVS wear a mask definitely influences me to second guess my decision to wear one.” (18) “Others practicing social distancing affects my ability more than my willingness to do so. I know it’s the best thing to do so I’m going to do it, but the more people do it the more it feels reassuring—that we are all in this together, that it’s less awkward the more normal it is, that we all want to take care of each other and ourselves. Even if others don’t do it I would want to set a good example.”
*Structural Dependence on Employer Policies and Institutional Support*
(19) “I’m more than willing to continue social distancing as long as I’m permitted to work from home.” (20) “The most important thing is that my employer continues to support us working from home. Everything else I can get by without.” (21) “The most important factor for me and those that I work with is the ability to continue working from home. This has worked out very well for the employees and the company, however my employer (and many other employers) seems to think that as soon as the city lifts the ban, there is NO need to continue allowing people to work from home, even though our business is not affected by allowing people to work from home. The owners do not see the “social value” in allowing people to continue working from home until there is adequate testing and or a vaccine.” (22) “We will social distance as long as we can work from home, until there is a treatment or the risk of spreading the virus is reduced and contact tracing is in place.” (23) “My ability to effectively socially distance has been dominated by my employer and choices that others have made. I’m fully committed to it, but I exist within many systems.” (24) “I work in a school, I don’t know how, if at all, social distancing will be able to be implemented. I worry there aren’t enough tests for us. Why risk catching it if you can’t be tested and treated?” (25) “At the end of the day, I am at the mercy of my employer. When they tell me I have to go back to the office and resume “normal” activities I will have to do it.” (26) “When I return to work is not under my control, it’s up to my employer so I hope that when we go back, they will have good practices in place.” (27) “I cannot social distance unless someone else is paying the bills. My job requires that I do not social distance. That is a danger to my clients and to myself, but I have to work to put the bills.”

## Data Availability

De-identified data supporting the findings of this study are available from the corresponding author upon reasonable request.

## Supplementary Materials

The following supporting information can be downloaded at: https://www.mdpi.com/article/10.3390/epidemiologia7020048/s1, Supplementary File S1: Survey Instrument; Supplementary File S2: Codebook and Code Summaries; Supplementary File S3: Multivariable logistic regression of respondent characteristics associated with rankings as highest priority.

## Abbreviations

The following abbreviations are used in this manuscript:

BIPOC	Black, Indigenous, and People of Color (American Indian or Alaskan Natives, Asian, Black, Hispanic, or LatinX)
USA	United States of America
