# Supplementary material for: Perceived Readiness and Ability to Socially Distance During the Early COVID-19 Epidemic in a U.S. Metropolitan Area: Implications for Local Public Health Preparedness
Source: Epidemiologia (Basel). 2026 Apr 2;7(2):48. doi: 10.3390/epidemiologia7020048 (PMC13115543; doi:10.3390/epidemiologia7020048)
Supplement: Supplementary file 1 [file epidemiologia-07-00048-s001.zip › Supplementary File S1.pdf]

COVID-19 STL Instrument

Start of Block: Information Sheet

Q1 We invite you to participate in a research study being conducted by investigators from Washington University in St. Louis to understand your opinions and behaviors related to the COVID-19 epidemic.

If you have questions for the research team, please contact Dr. Virginia McKay, virginia.mckay@wustl.edu or you may contact the Human Research Protection Office at 1-(800)-438-0445 or hrpo@wustl.edu.

Thank you very much for your consideration of this research study.

Page Break

Start of Block: Screening

Q2 In order to make sure that you meet our criteria and that you are the right person to participate in this survey, please answer the question below.

Q3 Are you 18 years or older?

- No, I am not (15)
- Yes, I am (16)

Skip To: End of Block If Are you 18 years or older? = Yes, I am

Skip To: Q4 If Are you 18 years or older? = No, I am not

Page Break

Start of Block: SECTION 1. Participant Characteristics

Q5
This first set of questions will ask about you and your background.

Q6 How old are you?

- 18-25 (1)
- 26-35 (2)
- 36-45 (3)
- 46-55 (4)
- 56-65 (5)
- 66-75 (6)
- 76-85 (7)
- 86 or older (8)

Q7 What is your 5-digit zip code where you live?

____

Q8 Do you live in St. Louis city or county, or somewhere else?

- ○ St. Louis City (1)
- ○ St. Louis County (2)
- ○ Another county or place in Missouri (3)
- ○ Another county or place in Illinois (4)
- ○ Other (5)

Q9 What is your ethnicity or race? (Please select all that apply)

- ▢ American Indian or Alaska Native (1)
- ▢ Asian (2)
- ▢ Black or African American (3)
- ▢ Hispanic or Latino (7)
- ▢ Native Hawaiian or other Pacific Islander (4)
- ▢ White (5)
- ▢ Other (6)

Q10 How do you describe yourself?

- ○ A man (1)
- ○ A woman (2)
- ○ Gender nonconforming / Other (3)
- ○ Prefer not to respond (4)

Q11 Including yourself, how many people live in your household?

▼ 1 (2) ... 10 or more (12)

Q12 Of the people that live in your household, how many are under 18?

▼ 0 (2) ... 10 or more (13)

Q13 Of the people that live in your household, how many are over 65?

▼ 0 (1) ... 10 or more (11)

Q14 Which of the following categories best describes your ANNUAL household income?
Annual household income is the income before taxes of *all persons* in your household *combined* for *one year*?

- ○ Less than $20,000 (1)
- ○ $20,000 to less than $30,000 (2)
- ○ $30,000 to less than $40,000 (3)
- ○ $40,000 to less than $50,000 (4)
- ○ $50,000 to less than $70,000 (5)
- ○ $70,000 to less than $100,000 (6)
- ○ $100,000 to less than $150,000 (7)
- ○ $150,000 or more (8)
- ○ Prefer not to respond (9)
- ○ I do not know (10)

Q15 Do you have any of the following conditions? (Please select all that apply)

- ▢ Asthma (1)
- ▢ Cancer in the past year (2)
- ▢ Chronic heart disease (High blood pressure, a recent heart attack, etc.) (3)
- ▢ Chronic kidney disease (4)
- ▢ Chronic lung disease (COPD, emphysema, etc.) (6)
- ▢ Diabetes (High sugar disease) (7)
- ▢ Immunosupressive condition (Lupus, HIV, etc.) (8)
- ▢ ⊗None of the above (9)

Q16 What type of health insurance do you currently have?

- ☐ No insurance/Self-pay (1)
- ☐ Employer-sponsored disability insurance (2)
- ☐ Medicaid (3)
- ☐ Medicare (4)
- ☐ National Health Insurance (5)
- ☐ Private or group health insurance (6)
- ☐ Veterans Affairs/Military (7)
- ☐ Unknown (8)
- ☐ Other, please specify (9) ____

Q17 Are you living in an area with a policy mandating social distancing measures (e.g., shelter-in-place or safer-at-home orders)?

- ○ No (3)
- ○ Yes (4)
- ○ I don't know (5)

Q18 Are you currently employed?

- ○ No (11)
- ○ Yes (12)
- ○ Yes, but I've been furloughed (13)

Skip To: Q19 If Are you currently employed? = Yes

Q21 Were you furloughed or laid off because of your employer’s response to the COVID-19 (the coronavirus) epidemic?

- No (1)
- Yes (2)

Display This Question:

If Are you currently employed? = Yes

Q19 Are you currently working from home?

- No, I'm still working in my office or business (1)
- Yes, I'm working from home (2)

Display This Question:

If Are you currently employed? = Yes

Q20 For your work, do you have to interact with customers face to face?

- No (1)
- Yes (2)

Page Break

Start of Block: SECTION 3. Perceptions of COVID-19

Q22 **The following questions will ask about your perceptions of COVID-19 (the coronavirus) and social distancing.**

Q23 In the past 30 days, have you had any of the following symptoms? (Please select as many as apply)

- ☐ Fever (1)
- ☐ Dry Cough (2)
- ☐ New or worsened shortness of breath (3)
- ☐ Runny nose (4)
- ☐ Itchy/watery eyes (5)
- ☐ Diarrhea or vomiting (6)
- ☐ Loss of sense of smell or taste (7)
- ☐ Intense fatigue (8)
- ☐ Body aches (9)
- ☐ I have not had any of these symptoms (10)

Q24 Have you had any symptoms that made you believe you had COVID-19 (including the ones checked above or others)?

- ○ Yes (1)
- ○ No (2)

Q25 Have you been tested for COVID-19?

- Yes (1)
- No (2)

Display This Question:

If Have you been tested for COVID-19? = No

Q26 Have you been diagnosed with COVID-19?

- Yes (1)
- No (2)

Display This Question:

If Have you been tested for COVID-19? = No

Q27 Would you like to be tested for COVID-19?

- Yes (1)
- No (2)

Q28 Within the next three months, how likely do you think you are to...

	Extremely unlikely (1)	Somewhat unlikely (2)	Neither likely nor unlikely (3)	Somewhat likely (4)	Extremely likely (5)
get COVID-19? (1)	○	○	○	○	○
be hospitalized because of COVID-19? (2)	○	○	○	○	○
practice social distancing? (3)	○	○	○	○	○

Q29 To what extent do you agree or disagree with the following statements?

	Strongly disagree (1)	Somewhat disagree (2)	Neither agree nor disagree (3)	Somewhat agree (4)	Strongly agree (5)
I am knowledgeable about social distancing (1)	○	○	○	○	○
I am able to practice social distancing (2)	○	○	○	○	○
Social distancing will help prevent me from getting COVID-19 (3)	○	○	○	○	○
Social distancing is easy to do (4)	○	○	○	○	○

Page Break

Start of Block: SECTION 4 Qualitative Questions

Q30 In one or more sentences, please describe how the COVID-19 epidemic has impacted you.

____

____

____

Start of Block: SECTION 2. Factors influencing Social Distancing

Q31 **We would like to know more about how you practice social distancing**.

By social distancing we mean deliberate actions to minimize contact with other people and reduce the spread of COVID-19 (the coronavirus).

Q32 What do you do when practicing social distancing? (Please select all that apply)

▢ I maintain a physical distance from other individuals (1)

▢ I avoid groups of people (2)

▢ I minimize trips requiring me to leave my home (3)

▢ I minimize visits from other people to my home (4)

▢ I wear protective gear like masks and gloves (5)

▢ I wash my hands after being in public (6)

▢ I sanitize purchased goods (7)

▢ I avoid touching my face, mouth, and nose (8)

▢ None of the above (9)

▢ Other (please specify) (10) ____

Q33 Starting today, for how long would you be willing to engage in the following behaviors?

	Not at all (1)	1-2 weeks (2)	3-4 weeks (3)	5-6 weeks (4)	7-8 weeks (5)	9 or more weeks (6)
Maintain a physical distance from other individuals (1)	○	○	○	○	○	○
Avoid groups of people (2)	○	○	○	○	○	○
Minimize trips requiring you to leave your home (3)	○	○	○	○	○	○
Minimize visits from other people to your home (4)	○	○	○	○	○	○
Wear protective gear like masks and gloves (5)	○	○	○	○	○	○
Wash your hands after being in public (6)	○	○	○	○	○	○
Sanitize purchased goods (7)	○	○	○	○	○	○
Avoid touching your face, mouth, and nose (8)	○	○	○	○	○	○

Q34 Below you will see a list of items that may impact your ability or willingness to practice social distancing **related to you and your relationship with others**. Please read through the items and then select the options that are *most* important or relevant for your concerns around social distancing.

- ▢ My own personal risk of getting COVID-19 (6)
- ▢ It feels socially awkward (7)
- ▢ Working from home (8)
- ▢ Continuing to work outside my home (9)
- ▢ Needing to leave for healthcare or to buy things I need (10)
- ▢ Having groceries and goods delivered (11)
- ▢ Having enough money to make bulk purchases (12)
- ▢ Having supplies like disinfecting wipes or hand sanitizer (13)
- ▢ A major life event like moving or a family death (14)
- ▢ Caring for others that are important to me (15)
- ▢ Caring for pets (16)
- ▢ My family and friends practicing social distancing (17)
- ▢ Having video chat and internet to interact with others remotely (18)
- ▢ Wanting to interact with others in person (i.e., friends, romantic and sexual partners, other family members) (19)

Carry Forward Selected Choices from "Below you will see a list of items that may impact your ability or willingness to practice social distancing related to you and your relationship with others. Please read through the items and then select the options that are most important or relevant for your concerns around social distancing."

Q35 You will see the factors that you selected below. Please rank them in order of importance for your ability and willingness to practice social distancing by dragging and dropping them, with the first item being the most important and the last item being the least important.
______ My own personal risk of getting COVID-19 (1)
______ It feels socially awkward (2)
______ Working from home (3)
______ Continuing to work outside my home (4)
______ Needing to leave for healthcare or to buy things I need (5)
______ Having groceries and goods delivered (6)
______ Having enough money to make bulk purchases (7)
______ Having supplies like disinfecting wipes or hand sanitizer (8)
______ A major life event like moving or a family death (9)
______ Caring for others that are important to me (10)
______ Caring for pets (11)
______ My family and friends practicing social distancing (12)
______ Having video chat and internet to interact with others remotely (13)
______ Wanting to interact with others in person (i.e., friends, romantic and sexual partners, other family members) (14)

Q36 Below you will see a list of items that may impact your ability or willingness to practice social distancing related to the COVID-19 epidemic and your community. Please read through the items and then select the options that are most important or relevant for your concerns around social distancing.

- ▢ The severity of the COVID-19 epidemic where I live or work (15)
- ▢ The availability of testing for COVID-19 where I live or work (16)
- ▢ The availability of treatment for COVID-19 (17)
- ▢ Clarity about how to practice social distancing (18)
- ▢ Evidence showing that social distancing is working (19)
- ▢ Businesses’ ability to ensure others practice social distancing and sanitation. (6)
- ▢ Businesses offering curbside pick up or delivery (7)
- ▢ Others practicing social distancing in public spaces, like parks or grocery stores (8)
- ▢ What others are saying on social media about COVID-19 and social distancing (9)
- ▢ Using public transportation (10)
- ▢ My employer having policies to support social distancing (11)
- ▢ Legal consequences of not practicing social distancing (12)
- ▢ Public assistance to help cover my needs (29)

Carry Forward Selected Choices from "Below you will see a list of items that may impact your ability or willingness to practice social distancing related to the COVID-19 epidemic and your community. Please read through the items and then select the options that are most important or relevant for your concerns around social distancing."

Q37 You will see the factors that you selected below. Please rank them in order of importance for your ability and willingness to practice social distancing by dragging and dropping them with the first item being the most important and the last item being the least important.

______ The severity of the COVID-19 epidemic where I live or work (1)
______ The availability of testing for COVID-19 where I live or work (2)
______ The availability of treatment for COVID-19 (3)
______ Clarity about how to practice social distancing (4)
______ Evidence showing that social distancing is working (5)
______ Businesses’ ability to ensure others practice social distancing and sanitation. (6)
______ Businesses offering curbside pick up or delivery (7)
______ Others practicing social distancing in public spaces, like parks or grocery stores (8)
______ What others are saying on social media about COVID-19 and social distancing (9)
______ Using public transportation (10)
______ My employer having policies to support social distancing (11)
______ Legal consequences of not practicing social distancing (12)
______ Public assistance to help cover my needs (13)

Q38 If there is anything you would like to explain about why you have chosen the above items, please do so in the space provided below.

____

____

____

____

____

Q39 Are there any other major factors impacting you that we haven’t listed here?

____

____

____

____

____

Page Break

Start of Block: Section 4 Health Communication

Q40 **This last section will ask questions about your perceptions of how COVID-19 information is communicated.**

Q41 In general, how much would you trust information about health or medical topics about COVID-19 from each of the following sources?

	Not at all (1)	A little (2)	A moderate amount (3)	A lot (4)
A doctor (1)	o	o	o	o
Family or friends (2)	o	o	o	o
Federal government health agencies (3)	o	o	o	o
State or local government health agencies (8)	o	o	o	o
Community organizations (4)	o	o	o	o
National political leaders (9)	o	o	o	o
State or local political leaders (10)	o	o	o	o
Religious organizations & leaders (5)	o	o	o	o
Internet Sources (6)	o	o	o	o
Social Media (7)	o	o	o	o

Q42 Imagine that you had a strong need to get information about COVID-19. Where would you go first?

- ○ Books (1)
- ○ Brochures, pamphlets, etc. (2)
- ○ Complementary, alternative, or unconventional practitioner (3)
- ○ Doctor or health care provider (4)
- ○ Family (5)
- ○ Friend/Co-worker (6)
- ○ Internet (7)
- ○ Library (8)
- ○ Magazines (9)
- ○ Newspapers (10)
- ○ Social Media (11)
- ○ Telephone information number (12)
- ○ Other (please specify) (13) ____

Start of Block: End of Survey

Q43 Thank you for completing the survey. We value your input as a way to inform our response to the COVID-19 epidemic.

If you have any questions or concerns, please don't hesitate to contact Dr. Virginia McKay by email at virginia.mckay@wustl.edu

Q44
If you are interested in participating in future work related to COVID-19, please leave your email address or telephone number below.

- ○ Email address (1) ____
- ○ Email address (for verification) (2) ____
- ○ Phone number (XXX) XXX-XXXX (3) ____
