# Supplementary material for: Perceived Readiness and Ability to Socially Distance During the Early COVID-19 Epidemic in a U.S. Metropolitan Area: Implications for Local Public Health Preparedness
Source: Epidemiologia (Basel). 2026 Apr 2;7(2):48. doi: 10.3390/epidemiologia7020048 (PMC13115543; doi:10.3390/epidemiologia7020048)
Supplement: Supplementary file 1 [file epidemiologia-07-00048-s001.zip › Supplementary File S2.pdf]

**Codebook and Code Summaries**

Research question: Which factors affect people's perceived readiness and ability to practice social distancing as a preventative behavior for COVID-19 in St. Louis, MO.

| Code | Definition | Example |
|---|---|---|
| **Severity** | Any discussion about how the severity of the epidemic in the community has influenced perceptions of the disease and social distancing | *"My area has been affected less than surrounding areas, which I hope will continue. If it does continue and more effective treatments for COVID 19 are developed, I would feel more confident in slowly lessening social distancing over time."* |
| **Availability of Testing and Treatment** | Discussion of how testing and treatment (the presence or lack of) influences social distancing behavior organization evaluates program, what they consider evidence for success | *"Testing is the single most important factor for our family to begin to drop our social distancing protocol. Next would be treatment. Even if stay at home orders are not extended, we do not intend to modify our behavior."*<br><br>*"Treatment for Covid is another thing that could reduce the risk of disease enough that, even before we understand the spread completely, we could feel safer being around other humans."* |
| **Social Distancing in public spaces** | How social distancing behaviors are influenced by others practicing it or not, in public spaces | *"I am very disturbed when I see groups of people together and not wearing masks. I feel that everyone should cover their faces when out in public."* |
| **Social Distancing and Workplace** | How measures put in place by employers' impact social distancing behaviors<br><br>Includes discussions on how being able to work from home or not may impact a person's social distancing behavior | *"PPE. I work in a non-Covid healthcare setting and have been working during stay at home orders. My employer put many people not on the frontlines at risk by reducing access to PPE that would have normally been used. As a result co-workers are on ventilators now. Fighting for proper PPE has been, by far, the most stressful part of this pandemic for me.."*<br><br>*"If we can do our jobs well while working remotely, than I don't see why we can't continue until there is treatment for COVID-19."* |
| **Evidence** | Discussion on how the perception of evidence behind social distancing influences preventative behavior | *"Evidence is important to me, but I don't need additional evidence that it works at this point."* |
| **Caring for Self and others** | Discussions of how caring and being responsible for others may impact social distancing behaviors<br><br>Also includes discussions of how an individual's perception on their disease risk influences social distancing behavior | *"I would add that concern about my immediate family play a large part in my social distancing choices, esp. my difficulty of social distancing when I return to work. There wasn't really a question that applied to that."*<br><br>*My greatest issues is trust i have vulnerability above others for covid infection and Iâ€™m willing to do whatever at all costs to reduce the risk of transmission.* |

<u>Availability of Testing and Treatment</u>

Majority of respondents expressed that the availability of testing and treatment in the region was a major factor influencing their willingness to social distance. Although factors relating to testing and treatment were explored separately, responses highlighted their interconnection. People expressed that they will be more willing to relax social distancing once they were able to get tested or receive a vaccination for the disease. Some particularly expressed the rationale that, once testing is widely available, and majority of the population gets tested, people will become aware of their risk or carrier status and this could inevitably decide who should continue social distancing and who should not, as people would eventually get tired of social distancing. Respondents said the following:

- Until there is sufficient, accurate testing & treatment for COVID, I am willing to stay home & help flatten the curve. There is no need to add to the overwhelming task that our frontline workers are currently dealing with.
- There must be more testing and ability to monitor those that have been exposed. There must be a vaccine very soon or we will have herd immunity the old fashion way... people won't keep up the social distancing much longer. The mental impact is real!
- We need better testing and treatment. The stay-at-home orders need to end soon. People will stop following if some restrictions arenâ€™t loosened
- I will practice social distancing as long as there is no vaccine and no proven treatment. I wish masks were mandated. They are most effective if everyone wears them. I feel like I am helping others by wearing one but very few people walking in the recently reopened parks are wearing them.

To others, this need for testing and treatment meant more to people's willingness to social distance, than recommendations made by public health authorities.

- Testing is the single most important factor for our family to begin to drop our social distancing protocol. Next would be treatment. Even if stay at home orders are not extended, we do not intend to modify our behavior.
- It seems like widespread testing for illness and antibodies would give us a true sense of the severity and spread of Covid; without widespread testing we are really making decisions based on very incomplete and possibly misleading data. Testing also would allow those with antibodies and those without current illness to go to work/shop/etc, while those who test positive could be isolated and traced.
- I don't trust easing of social distancing measures if we don't have adequate testing and treatment in place.

Respondent also generally expressed their concerns about the lack of testing availability and the financial costs associated with getting tested:

- No one has notified of any testing available and we don't know who to call. I am over 63 years and my husband is 78. Hearing about the severity of symptoms I am concerned about getting the virus. I believe in the "right to try". I have heard a person call in to the local radio station, of being sent home and not treated for the illness, only to have to return to the emergency room because the he could not get his breath. Ultimately he was treated with hydroxichloroquin (SP) and recovered. He is younger than I am. That vaccine can't come fast enough. We have children and grand children we would like to visit (on each coast) but are afraid to travel.
- I cannot social distance unless someone else is paying the bills. My job requires that I do not social distance. That is a danger to my clients and to myself, but I have to work to put the bills. They are also not testing many people, only if you seem to be on the brink of death. If I have symptoms, I have to be cleared by a doctor for the flu before they will even screen me for Covid-19. I have tried.

Evidence

People were willing to practice social distancing if they were convinced that such preventative behaviors were effective in reducing the spread of the disease and protecting the population. Majority of respondents felt that social distancing was working effectively to reduce the viral spread.

- To me the fact that social distancing is working keeps me happier to continue it . It brings back a little bit of normal to be able to pick up food from some of our favorite restaurants. Living in a community where most people are practicing. social distancing makes it easier for everyone. You can feel safe to go for a walk or not feel crowded if you have to stop at a store. I donâ€™t feel that testing is readily available but it will need to be better before I will feel safe going out.
- I believe in the science and logic of social distancing and the health and safety of myself, my family and those in my community is important to me; thus, we have made significant changes to our life to achieve social distancing. While we practice this, we try to cautiously support local businesses through curb side pick up and we limit our trips to the grocery store.
- I know that social distancing is working, but I feel is is very important for others to see that evidence. And we need universal testing, it near-universal. We aren't finding cases because we aren't testing, especially in Black and low-income areas.
- Testing and tracking for Covid-19 would be so helpful in knowing where to be safe. Prevention is ideal. Treatment would save lives. I like to know the incidence in my area, so that I would just stay home if it feels high to me. That means no walks or groceries. For now, I think social distancing is key. Going out requires the understanding and cooperation of others. I feel safer with curbside pickup and support businesses that practice it.

When others doubted the evidence in a preventative behavior, they were less likely to continue that practice. For many respondents, the uncertainty around the disease and the lack of consistency in communication of evidence made it hard for them to practice social distancing:

- We need to figure out how much, if any, herd immunity is out there already. Many people I know think they may have already had it but the antibody tests are not trustworthy. If we can measure this we can 1) prove to folks they didn't already have it and need to practice social distancing 2) figure out where we are in the grand scheme of getting past this. I realize we don't know if we can be re-infected. This too also needs to be determined.
- Something that underlies all of this is consistency in information being provided and how much I trust the sources of information. Iâ€™m trying to both take the most conservative approach I can, without also being alarmist. So- I wear a mask while shopping, but I do not attempt to â€œsanitizeâ€ my purchases. I honestly donâ€™t know how much wearing a mask helps, but I have yet to be convinced to any extent that wasting lysol wipes on my box of cereal helps.
- It's frustrating not to know what is effective, and not to know if me, my friends, my family and coworkers can be safe from catching this. It is increasingly clear that social distancing is not a good solution because we cannot stop the spread of the virus no matter what we do (see South Korea). The toll on our economy will destroy millions of lives and kill scores of people in the third world. I can't stand for that. Therefore, I will not practice social distancing.
- Seems testing is available only if several symptom checkboxes are ticked. Worry is that if someone has COVID but is asymptomatic, then they could spread it, no symptoms encourages unfounded confidence - people who don't have symptoms may even stop social distancing thinking they're in the clear. Second, truthful and accurate reporting is needed - information distributed should have nothing to do with getting re-elected. Without more wide-spread TESTING, we don't know if it's really safe to re-open

events, travel, see family, etc. Without FACTS, people don't understand the need for continued social distancing.

- I get frustrated because physicians on the news will say things like, "It's *highly unlikely* you'll acquire the virus if you do not come into direct contact with a symptomatic person," and then the next week it's announced that asymptomatic spread is likely. I understand that this is a unique experience, but the feeling that we are not being told, "We do not know the right answer but this is what we currently think may be true and recommend," makes it difficult trust that public health recommendations are not being heavily tailored to reduce the potential for public panic instead of reducing the potential for covid transmission.

Additionally, for other respondents, the uncertainty of the disease rather made them willing to practice social distancing:

- At this point, it is hard to fathom what kind of data would need to come out to demonstrate that we are safe to stop social distancing - there seems to be too much unknown for me to feel comfortable not social distancing any time soon.
- I just want clear information on what is working and what is not, as well as clear guidelines, even if they seem "too cautious." I am someone who is able to distance/isolate a decent amount, but I want as exact of information as possible. I also hear that we are reopening things and just do not understand why. It feels premature and unsafe, so I want better explanations about that.

<u>Social Distancing by Others</u>

A vast majority of respondents expressed their concerns about how their social distancing behaviors were affected by other people in the community. Respondents explained that they were more inclined to shelter in place or avoid going out to get groceries for example, if they knew others would not be practicing effective social distancing.

- Iâ€™m much less likely to visit a business that doesnâ€™t seem to make social distancing a priority (employees not wearing masks or congregating in groups, little to no signage or encouragement to social distance, etc) for example I was going to order takeout from a local restaurant until I drove by and saw the employees on break all standing in a big group outside, no masks and much too close together
- What impacts my ability to perform social distancing is when I go to stores and employs are not wearing masks. Or customers (also not wearing masks) violate the 6â€™ distance rule. For instance, yesterday I went to Loweâ€™s for paint. It was packed. I wonâ€™t go back. Iâ€™ll order online instead. Even after some of the rules are lifted in the area, we will continue to frequent businesses that offer curb-side pick-up/delivery until treatments or a vaccine is available.
- I don't really trust other's dedication; I see incorrectly wearing of masks (nose exposed) so I would rather stay home than swim with idiots.
- I have not been in a grocery store for 7 weeks now because the last time I did it was terrifying. Blatant disregard for social distancing. I was running away from the old ladies FOR THEIR SAKE. And my dad works in a supermarket, so I'm scared to death for him. The fewer people coming & going in those doors, the better. We've been ordering carryout from restaurants about twice a week (probably a bit more than normal for us) because we want them to survive with us.
- I feel fairly comfortable with my ability to reduce my risk and that of my family; however between working outside the home and leaving the house for essential errands, I worry about the increased risk due to *other* people not following social distancing and good hygiene guidelines.

- I have no problem practicing social distancing myself, others make it difficult. Stores and police do not enforce the rules. People are rude and donâ€™t care and surely do not stay 6 feet away when in public. I have had to ask people to step away from me in stores.

For some respondents, there was a ‘peer-pressure’ aspect of practicing social distancing, especially with regards to mask wearing. Some people explained that they were likely to wear a mask in places where others wore mask. Consequently, such people were less inclined to wear a mask if others were not in masks

- I don't like being seen as hysterical so seeing no one else in CVS wear a mask definitely influences me to second guess my decision to wear one
- Others practicing social distancing" affects my ability more than my willingness to do so. I know it's the best thing to do so I'm going to do it, but the more people do it the more it feels reassuring - that we are all in this together, that it's less awkward the more normal it is, that we all want to take care of each other and ourselves. Even if others don't do it I would want to set a good example.
- If I have to go in to work, I can't do anything about that, I'd have to go. How much legal consequences would affect me would depend on how they were enforced- I don't see them being very easy to enforce, and if they were enforced intensely, it might come with a big backlash. Meanwhile, if all of my friends were practicing social distancing intensely, I would feel bad if I were to not social distance properly

Some respondents argued that a way to encourage others to practice effective social distancing is by having disciplinary measures in place for people who did not practice proper social distancing, while other also expressed that the enforcement of disciplinary actions could affect specific populations adversely in the case of biased enforcement.

- There are no legal repercussions for those not practicing social distancing so itâ€™s not forcing people to comply.
- Legal consequences sound appealing because of how vital it is that we all social distance, but they are low-priority for me because I am skeptical that they would be applied equally and fairly for all races/classes. E.g. in New York City there have been instances where white, middle-to-upper class people are offered masks by the police when they don't have them, but people of color and/or working-class people get citations

Lastly, there was a general consensus that people were not social distancing effectively because of poor health communication, and that people were not completely aware or convinced of the need for social distancing. Respondents explained that people needed to be educated on how to practice safe social distancing:

- I feel that it is important for people to know the reasons why social distancing is important and for those reasons to be supported both by the communities and the businesses. If they are not recognized by businesses AND communities, then it will be likely that people will not take things seriously

## Social Distancing and Workplaces

The ability to make an income while social distancing was an influential factor for social distancing among many respondents. Respondents who were working remotely were more willing and able to practice social distancing. People were confident that as long as they continued to work from home they will be able to practice social distancing:

- Iâ€™m more than willing to continue social distancing as long as Iâ€™m permitted to work from home.
- The most important thing is that my employer continue to support us working from home. Everything else I can get by without

- The most important factor for me and those that I work with is the ability to continue working from home. This has worked out very well for the employees and the company, however my employer (and many other employers) seem to think that as soon as the city lifts the ban, there is NO need to continue allowing people to work from home, even though our business is not affected by allowing people to work from home. The owners do not see the "social value" in allowing people to continue working from home until there is adequate testing and or a vaccine.
- If i wasn't employed, couldn't work from home, or didn't have a car that would decrease my ability to practice social distancing a lot.
- I would like to be able to continue working from home forever! I can be as effective, and there is no risk of me spreading the virus or interacting with others. I think all employers should offer this as an option. Those that can consistently work from home can help keep others safe.
- We will social distance as long as we can work from home, until there is a treatment or the risk of spreading the virus is reduced and contact tracing is in place.

Some respondents who were required to go to a workplace expressed doubts about their ability to social distance. Most of them felt their ability to social distance ultimately depended on their employers decisions and their place of employment i.e., their colleagues might not practice effectively or employers wouldn't promote a conducive social distancing environment

- I work in a school, I don't know how, if at all, social distancing will be able to be implemented. I worry there aren't enough tests for us. Why risk catching it if you can't be tested and treated?
- Iâ€™m a 5th grade teacher and want to return to class but not sure how we can practice social distancing at school.
- My employer may be able to enforce social distancing with our customers at the restaurant when we reopen our dining room (although has not given us a plan for it yet), but we have not been able to practice social distancing in the kitchen and front line of our restuarant. We know not all of our employees actually follow social distancing guidelines outside of the workplace and have not always followed the stay at home order. It's worrisome that we could've been exposed to the virus just because an employee starting dating someone new or visited friends/family during the pandemic.
- My ability to effectively socially distance has been dominated by my employer and choices that others have made. I'm fully committed to it, but I exist within many systems.
- At the end of the day I am at the mercy of my employer. When they tell me I have to go back to the office and resume "normal" activities I will have to do it.
- When I return to work is not under my control, itâ€™s up to my employer so I hope that when we go back, they will have good practices in place.

Although generally people feared to lose their jobs if they decided to shelter in place and engage in preventative behaviors, others expressed their willingness to forgo their jobs to be able to effectively social distance. Below are some examples of what people said concerning their willingness to work in places where social distancing was not effectively practiced.

- I cannot social distance unless someone else is paying the bills. My job requires that I do not social distance. That is a danger to my clients and to myself, but I have to work to put the bills.
- I avoid stores where most people don't follow the current rules. And if school districts insist on piling 30 students into a classroom next fall I will not teach in the buildings

Respondents also expressed that they were able to practice social distancing because they received a form of financial or social support services from the government.

- I am able to stay home because I am getting unemployment insurance and the $600 weekly federal unemployment benefit. Without those, I would need to work to have money to live
- It's not safe until we have a treatment for covid-19. My family is fortunate enough to be able to work from home and receive unemployment insurance and funds from the CARES Act. We can afford to pay for deliveries of goods to our home.

<u>Severity of the disease</u>

The severity of the disease in the region was a motivating factor for respondents to practice social distancing. Some respondents shared the number of deaths and the rate of spread were concerning and had made them more cautious.

- There seems to be a difference between the things that make social distancing absolutely imperative vs the things that make social distancing more comfortable. Like, there severity of the outbreak and the availability of treatment are critical factors, whereas the availability of curbside just makes it emotionally easier.
- My willingness and ability to practice social distancing is increased by the aforementioned factors. COVID-19 is spreading quickly in the St. Louis area and I donâ€™t want to get or be a carrier so I stay home other than going to work. At work and the grocery store I wear a mask, use hand sanitizer until Iâ€™m able to wash my hands, and stay at least 6ft away from others.
- I donâ€™t think some people are taking this situation as seriously as they should and arenâ€™t following the precautions they should. If I saw that the severity of the outbreak in my area was improving, I would start to relax the precautions Iâ€™m taking, but not totally.
- I think the severity of the outbreak, and how easy it is to get tested and treated would make me more cautious, but even a small outbreak could spread quickly without controls--mainly distancing. If there is a strong, clear, consistent message that everyone understands and I see people following it when I am out--basically top-down orders--I can see myself doing what I am told to do much better, sad to say. Guess I am a follower.
- Number of deaths daily in the city of St. Louis is concerning.
- Great number of people not practicing social distancing, mask wearing etc is concerning.
- The lack of testing of more people is concerning.
- The ease of businesses switching to curbside and takeout/online ordering needs to increase.

<u>Caring for Self or Others</u>

People were willing to practice social distancing when they perceived themselves or close relations to have a higher risk of experiencing unfavorable outcomes from the disease.

- My greatest issues is trust i have vulnerability above others for covid infection and Iâ€™m willing to do whatever at all costs to reduce the risk of transmission.
- I know what do do, and I'm willing to keep social distance to protect others, especially my wife who is at a high risk for complications. But I worry about the lack of concern of people who protest that these measures impose on their rights. They fail to realize their responsibility to maintain health in the community, and they scare me because they don't care for anyone other than themselves.
- My daughter is a type one diabetic, we are very motivated to keep her away from the virus until a better treatment is available
- I just don't want to go back and work in a clinic where staff and patients are not wearing masks and we work in a small room. This terrifies me. My mom is going through chemo and I need to visit her/bring

groceries - I'm staying far away but this is priority and I'd never forgive myself if i brought her something.

- I am very concerned about other people getting my family sick, and us spreading it to my family member with stage 4 cancer. He lives alone so my husband is one of his main caregivers. We have no choice other than to take the most precautions necessary.
- I will continue to social distance because I donâ€™t want to be a vector for disease or the reason someone else has to suffer. I also donâ€™t want to experience potential irreversible organ damage or heart failure.
- Frankly, *everything* you listed is important to me, to one degree or another.
  The honest truth is that I will keep practicing social distancing (and keeping in touch with my therapist) because my family and friends are too important to me to risk by letting restrictions relax. It sucks, a lot, and I really do hate this all, but it doesn't change the fact that it's necessary and must be done.
