# Supplementary material for: Perceived Readiness and Ability to Socially Distance During the Early COVID-19 Epidemic in a U.S. Metropolitan Area: Implications for Local Public Health Preparedness
Source: Epidemiologia (Basel). 2026 Apr 2;7(2):48. doi: 10.3390/epidemiologia7020048 (PMC13115543; doi:10.3390/epidemiologia7020048)
Supplement: Supplementary file 1 [file epidemiologia-07-00048-s001.zip › Supplementary File S3.pdf]

Supplementary File S3.

Multivariable logistic regression of respondent characteristics associated with rankings as highest priority

Supplementary Table S1. Multivariable logistic regression of respondent characteristics associated with ranking "My own personal risk of getting COVID" as highest priority

Predictor	aOR	95% CI	p
Age 46+ (vs 18–45)	1.62	1.18–2.22	**0.0028***
Men (vs Women)	1.11	0.76–1.60	0.4180
Gender nonconforming (vs Women)	0.85	0.24–2.35	0.7744
White alone (vs BIPOC)	0.61	0.41–0.92	**0.0156***
Income $40–70k (vs <$40k)	1.43	0.87–2.39	0.1554
Income $70–100k (vs <$40k)	1.64	0.99–2.74	0.0573
Income ≥$100k (vs <$40k)	1.48	0.93–2.39	0.1016
Cancer/Immunosuppressive (vs reference comorbidity group)	1.09	0.46–2.48	0.8459
Cardiovascular (vs reference comorbidity group)	0.75	0.37–1.52	0.4290
None of the above (vs reference comorbidity group)	0.48	0.30–0.77	**0.0018***
Other conditions (vs reference comorbidity group)	1.02	0.57–1.84	0.9430
Currently unemployed (vs reference work group)	1.49	0.95–2.37	0.0827
Working from home (vs reference work group)	0.96	0.63–1.47	0.8337

* Statistical significance ($p < 0.05$) is indicated in bold.

Supplementary Table S2. Multivariable logistic regression of respondent characteristics associated with ranking "Caring for others that are important to me" as highest priority

Predictor	aOR	95% CI	p
Age 46+ (vs 18–45)	1.59	1.12–2.27	**0.0097***
Men (vs Women)	0.85	0.55–1.32	0.4735
Gender nonconforming (vs Women)	0.60	0.14–2.60	0.4902
White alone (vs BIPOC)	1.45	0.84–2.51	0.1797
Income $40–70k (vs <$40k)	1.16	0.65–2.09	0.6146
Income $70–100k (vs <$40k)	0.95	0.52–1.75	0.8729
Income ≥$100k (vs <$40k)	1.38	0.81–2.35	0.2294
Cancer/Immunosuppressive (vs reference comorbidity group)	0.92	0.31–2.74	0.8762
Cardiovascular (vs reference comorbidity group)	0.75	0.29–1.91	0.5427
None of the above (vs reference comorbidity group)	1.14	0.64–2.02	0.6519
Other conditions (vs reference comorbidity group)	0.76	0.35–1.67	0.5006
Currently unemployed (vs reference work group)	0.92	0.53–1.58	0.7545
Working from home (vs reference work group)	1.35	0.84–2.17	0.2113

* Statistical significance ($p < 0.05$) is indicated in bold.

Supplementary Table S3. Multivariable logistic regression of respondent characteristics associated with ranking "Working from home" as highest priority

Predictor	aOR	95% CI	p
Age 46+ (vs 18–45)	0.56	0.35–0.89	**0.0137***
Men (vs Women)	0.78	0.47–1.30	0.3402
Gender nonconforming (vs Women)	2.61	0.97–7.03	0.0570
White alone (vs BIPOC)	0.65	0.39–1.08	0.0990
Income $40–70k (vs <$40k)	1.45	0.71–2.92	0.3055
Income $70–100k (vs <$40k)	1.84	0.92–3.70	0.0871
Income ≥$100k (vs <$40k)	1.68	0.88–3.22	0.1172
Cancer/Immunosuppressive (vs reference comorbidity group)	0.16	0.02–1.27	0.0830
Cardiovascular (vs reference comorbidity group)	1.70	0.62–4.68	0.3067
None of the above (vs reference comorbidity group)	1.07	0.56–2.05	0.8279
Other conditions (vs reference comorbidity group)	0.95	0.38–2.36	0.9120
Currently unemployed (vs reference work group)	7.43	0.95–57.91	0.0556
Working from home (vs reference work group)	41.36	5.73–298.74	**0.0002***

* Statistical significance (p < 0.05) is indicated in bold.

Supplementary Table S4. Multivariable logistic regression of respondent characteristics associated with ranking "The severity of the COVID-19 epidemic where I live or work" as highest priority

Predictor	aOR	95% CI	p
Age 46+ (vs 18–45)	0.93	0.60–1.46	0.7620
Men (vs Women)	1.66	1.06–2.62	**0.0278***
Gender nonconforming (vs Women)	0.83	0.19–3.67	0.8038
White alone (vs BIPOC)	0.53	0.32–0.87	**0.0127***
Income $40–70k (vs <$40k)	0.64	0.33–1.26	0.1991
Income $70–100k (vs <$40k)	0.82	0.43–1.56	0.5400
Income ≥$100k (vs <$40k)	0.88	0.50–1.56	0.6688
Cancer/Immunosuppressive (vs reference comorbidity group)	0.86	0.22–3.37	0.8318
Cardiovascular (vs reference comorbidity group)	1.20	0.42–3.43	0.7385
None of the above (vs reference comorbidity group)	1.02	0.51–2.05	0.9632
Other conditions (vs reference comorbidity group)	0.73	0.28–1.94	0.5324
Currently unemployed (vs reference work group)	1.44	0.74–2.78	0.2792
Working from home (vs reference work group)	1.31	0.72–2.38	0.3804

* Statistical significance (p < 0.05) is indicated in bold.

Supplementary Table S5. Multivariable logistic regression of respondent characteristics associated with ranking "The availability of testing for COVID-19 where I live or work" as highest priority

Predictor	aOR	95% CI	p
Age 46+ (vs 18–45)	0.82	0.57–1.18	0.2823
Men (vs Women)	0.85	0.56–1.29	0.4558
Gender nonconforming (vs Women)	0.60	0.17–2.06	0.4142
White alone (vs BIPOC)	1.07	0.66–1.75	0.7812
Income $40–70k (vs <$40k)	1.06	0.60–1.88	0.8426
Income $70–100k (vs <$40k)	1.70	0.98–2.92	0.0568
Income ≥$100k (vs <$40k)	1.25	0.75–2.09	0.3874
Cancer/Immunosuppressive (vs reference comorbidity group)	1.40	0.48–4.05	0.5338
Cardiovascular (vs reference comorbidity group)	2.00	0.84–4.75	0.1183
None of the above (vs reference comorbidity group)	1.49	0.80–2.75	0.2051
Other conditions (vs reference comorbidity group)	1.04	0.45–2.37	0.9288
Currently unemployed (vs reference work group)	1.08	0.66–1.78	0.7602
Working from home (vs reference work group)	0.91	0.59–1.42	0.6885

Supplementary Table S6. Multivariable logistic regression of respondent characteristics associated with ranking "The availability of treatment for COVID-19" as highest priority

Predictor	aOR	95% CI	p
Age 46+ (vs 18–45)	1.05	0.73–1.52	0.7859
Men (vs Women)	0.97	0.63–1.49	0.8938
Gender nonconforming (vs Women)	1.17	0.38–3.58	0.7793
White alone (vs BIPOC)	1.65	0.93–2.93	0.0892
Income $40–70k (vs <$40k)	1.54	0.87–2.73	0.1406
Income $70–100k (vs <$40k)	0.95	0.52–1.76	0.8821
Income ≥$100k (vs <$40k)	1.35	0.79–2.31	0.2756
Cancer/Immunosuppressive (vs reference comorbidity group)	0.80	0.27–2.38	0.6886
Cardiovascular (vs reference comorbidity group)	1.09	0.46–2.57	0.8525
None of the above (vs reference comorbidity group)	0.80	0.46–1.41	0.4449
Other conditions (vs reference comorbidity group)	0.99	0.47–2.06	0.9732
Currently unemployed (vs reference work group)	1.75	1.02–2.99	**0.0423***
Working from home (vs reference work group)	1.21	0.74–2.00	0.4473

* Statistical significance ($p < 0.05$) is indicated in bold.
